# Aqua­{6,6′-dieth­oxy-2,2′-[ethane-1,2-diyl­bis(nitrilo­methanylyl­idene)]diphen­olato}zinc

**DOI:** 10.1107/S1600536811031497

**Published:** 2011-08-11

**Authors:** Chen-Yi Wang, Xiang Wu, Juan-Juan Hu, Zhi-Ping Han

**Affiliations:** a Department of Chemistry, Huzhou University, Huzhou 313000, People’s Republic of China; b Huzhou No. 11 Middle School, Huzhou 313000, People’s Republic of China

## Abstract

The mononuclear zinc title complex, [Zn(C_20_H_22_N_2_O_4_)(H_2_O)], was obtained by the reaction of 3-eth­oxy­salicyl­aldehyde, ethane-1,2-diamine, and zinc acetate in methanol. The Zn atom is five-coordinated by two phenolate O and two imine N atoms of the tetradentate Schiff base ligand and by one water O atom, forming a square-pyramidal geometry. In the crystal, pairs of mol­ecules are linked *via* inter­molecular O—H⋯O hydrogen bonds, forming dimers.

## Related literature

For Schiff base complexes reported by our group, see: Wang (2009 ▶); Wang & Ye (2011 ▶). For similar zinc complexes, see: Meyer & Roesky (2007 ▶); Chu *et al.* (2008 ▶); Szlyk *et al.* (2005 ▶); Reglinski *et al.* (2002 ▶).
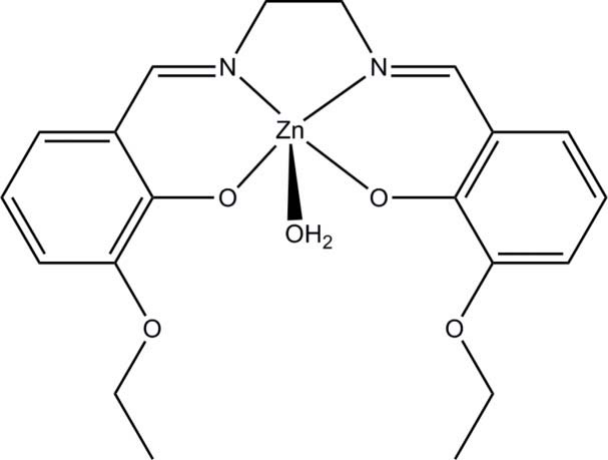


## Experimental

### 

#### Crystal data

[Zn(C_20_H_22_N_2_O_4_)(H_2_O)]
*M* *_r_* = 437.78
Monoclinic, 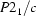

*a* = 13.545 (3) Å
*b* = 11.550 (2) Å
*c* = 14.327 (3) Å
β = 115.656 (3)°
*V* = 2020.4 (7) Å^3^
*Z* = 4
Mo *K*α radiation
μ = 1.25 mm^−1^
*T* = 298 K
0.23 × 0.20 × 0.20 mm

#### Data collection

Bruker SMART CCD area-detector diffractometer
Absorption correction: multi-scan (*SADABS*; Sheldrick, 1996 ▶) *T*_min_ = 0.762, *T*_max_ = 0.788
10161 measured reflections
3724 independent reflections
2607 reflections with *I* > 2σ(*I*)
*R*_int_ = 0.045

#### Refinement

*R*[*F* ^2^ > 2σ(*F* ^2^)] = 0.037
*wR*(*F* ^2^) = 0.085
*S* = 1.01
3724 reflections
261 parameters
3 restraints
H atoms treated by a mixture of independent and constrained refinement
Δρ_max_ = 0.37 e Å^−3^
Δρ_min_ = −0.28 e Å^−3^

### 

Data collection: *SMART* (Bruker, 1998 ▶); cell refinement: *SAINT* (Bruker, 1998 ▶); data reduction: *SAINT*; program(s) used to solve structure: *SHELXS97* (Sheldrick, 2008 ▶); program(s) used to refine structure: *SHELXL97* (Sheldrick, 2008 ▶); molecular graphics: *SHELXTL* (Sheldrick, 2008 ▶); software used to prepare material for publication: *SHELXTL*.

## Supplementary Material

Crystal structure: contains datablock(s) global, I. DOI: 10.1107/S1600536811031497/hg5074sup1.cif

Structure factors: contains datablock(s) I. DOI: 10.1107/S1600536811031497/hg5074Isup2.hkl

Additional supplementary materials:  crystallographic information; 3D view; checkCIF report

## Figures and Tables

**Table 1:** Selected bond lengths (Å)

Zn1—O1	1.9737 (19)
Zn1—O2	1.9990 (18)
Zn1—O5	2.040 (2)
Zn1—N2	2.075 (2)
Zn1—N1	2.080 (2)

**Table 2:** Hydrogen-bond geometry (Å, °)

*D*—H⋯*A*	*D*—H	H⋯*A*	*D*⋯*A*	*D*—H⋯*A*
O5—H5*A*⋯O3^i^	0.85 (1)	2.41 (2)	3.128 (3)	143 (3)
O5—H5*A*⋯O1^i^	0.85 (1)	2.03 (2)	2.781 (3)	147 (3)
O5—H5*B*⋯O4^i^	0.84 (1)	2.42 (2)	3.104 (3)	139 (3)
O5—H5*B*⋯O2^i^	0.84 (1)	1.96 (2)	2.722 (2)	149 (3)

Symmetry code: (i) 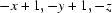.

**Fig. 1.**
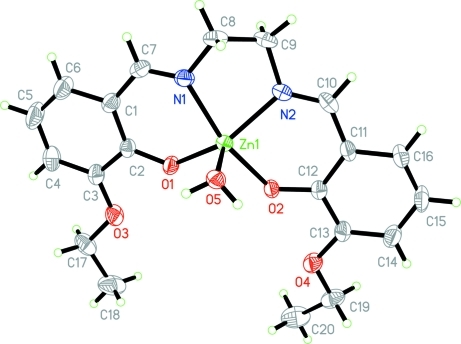
The molecular structure of the title compound, showing the atom-numbering scheme. Displacement ellipsoids are drawn at the 30% probability level.

**Fig. 2.**
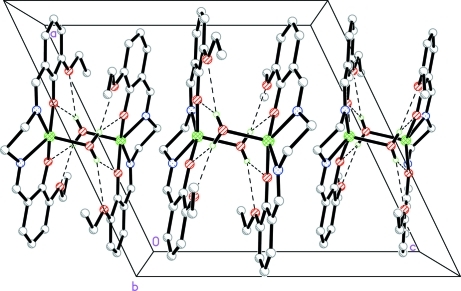
The molecular packing of the title compound, viewed along the a axis. Hydrogen bonds are drawn as dashed lines. Hydrogen atoms not related to the hydrogen bonding are omitted.

## supplementary crystallographic information

### Comment

As part of our investigations into Schiff base complexes (Wang & Ye, 2011; Wang, 2009), we have synthesized the title compound, a new mononuclear zinc(II) complex, Fig. 1. The Zn atom in the complex is five-coordinated by two phenolate O and two imine N atoms of the Schiff base ligand, and by one water O atom, forming a square pyramidal geometry. The Zn atom deviates from the least squares plane defined by the four basal donor atoms by 0.449 (2) Å. The Zn–O and Zn–N bond lengths (Table 1) are typical and are comparable with those observed in other similar zinc(II) complexes (Meyer & Roesky, 2007; Chu *et al.*, 2008; Szlyk *et al.*, 2005; Reglinski *et al.*, 2002).

In the crystal structure, adjacent two molecules are linked *via* intermolecular O—H···O hydrogen bonds, to form a dimer (Table 1, Fig. 2).

### Experimental

3-Ethoxysalicylaldehyde (1.0 mmol, 0.166 g) and ethane-1,2-diamine (0.5 mmol, 0.030 g) were dissolved in MeOH (30 ml), to the mixture was added with stirring an aqueous solution (5 ml) of zinc acetate dihydrate (0.5 mmol, 0.110 g). The final mixture was stirred at room temperature for 10 min to give a clear colorless solution. After keeping the solution in air for a week, colorless block-shaped crystals were formed at the bottom of the vessel.

### Refinement

The water H atoms were located from a difference Fourier map and refined isotropically, with O—H and H···H distances restrained to 0.85 (1) and 1.37 (2) Å. The remaining H atoms were placed in geometrically idealized positions and constrained to ride on their parent atoms, with C—H distances in the range 0.93–0.97 Å, and with *U*_iso_(H) set at 1.2*U*_eq_(C) and 1.5*U*_eq_(methyl C).

### Crystal data

**Table:**

[Zn(C_20_H_22_N_2_O_4_)(H_2_O)]	*F*(000) = 912
*M**_r_* = 437.78	*D*_x_ = 1.439 Mg m^−^^3^
Monoclinic, *P*2_1_/*c*	Mo *K*α radiation, λ = 0.71073 Å
Hall symbol: -P 2ybc	Cell parameters from 1989 reflections
*a* = 13.545 (3) Å	θ = 2.3–24.9°
*b* = 11.550 (2) Å	µ = 1.25 mm^−^^1^
*c* = 14.327 (3) Å	*T* = 298 K
β = 115.656 (3)°	Block, colorless
*V* = 2020.4 (7) Å^3^	0.23 × 0.20 × 0.20 mm
*Z* = 4	

### Data collection

**Table:**

Bruker SMART CCD area-detector diffractometer	3724 independent reflections
Radiation source: fine-focus sealed tube	2607 reflections with *I* > 2σ(*I*)
graphite	*R*_int_ = 0.045
ω scans	θ_max_ = 25.5°, θ_min_ = 2.4°
Absorption correction: multi-scan (*SADABS*; Sheldrick, 1996)	*h* = −16→16
*T*_min_ = 0.762, *T*_max_ = 0.788	*k* = −9→13
10161 measured reflections	*l* = −17→17

### Refinement

**Table:**

Refinement on *F*^2^	Primary atom site location: structure-invariant direct methods
Least-squares matrix: full	Secondary atom site location: difference Fourier map
*R*[*F*^2^ > 2σ(*F*^2^)] = 0.037	Hydrogen site location: inferred from neighbouring sites
*wR*(*F*^2^) = 0.085	H atoms treated by a mixture of independent and constrained refinement
*S* = 1.01	*w* = 1/[σ^2^(*F*_o_^2^) + (0.034*P*)^2^ + 0.0614*P*] where *P* = (*F*_o_^2^ + 2*F*_c_^2^)/3
3724 reflections	(Δ/σ)_max_ < 0.001
261 parameters	Δρ_max_ = 0.37 e Å^−^^3^
3 restraints	Δρ_min_ = −0.28 e Å^−^^3^

### Special details

**Table:**

Geometry. All e.s.d.'s (except the e.s.d. in the dihedral angle between two l.s. planes) are estimated using the full covariance matrix. The cell e.s.d.'s are taken into account individually in the estimation of e.s.d.'s in distances, angles and torsion angles; correlations between e.s.d.'s in cell parameters are only used when they are defined by crystal symmetry. An approximate (isotropic) treatment of cell e.s.d.'s is used for estimating e.s.d.'s involving l.s. planes.
Refinement. Refinement of *F*^2^ against ALL reflections. The weighted *R*-factor *wR* and goodness of fit *S* are based on *F*^2^, conventional *R*-factors *R* are based on *F*, with *F* set to zero for negative *F*^2^. The threshold expression of *F*^2^ > σ(*F*^2^) is used only for calculating *R*-factors(gt) *etc*. and is not relevant to the choice of reflections for refinement. *R*-factors based on *F*^2^ are statistically about twice as large as those based on *F*, and *R*- factors based on ALL data will be even larger.

### Fractional atomic coordinates and isotropic or equivalent isotropic displacement parameters (Å^2^)

**Table:**

	*x*	*y*	*z*	*U*_iso_*/*U*_eq_	
Zn1	0.49151 (3)	0.37501 (3)	0.10550 (2)	0.03753 (13)	
N1	0.3923 (2)	0.2318 (2)	0.08934 (18)	0.0452 (6)	
N2	0.5943 (2)	0.2758 (2)	0.23068 (18)	0.0443 (6)	
O1	0.35950 (15)	0.47362 (17)	0.05034 (14)	0.0437 (5)	
O2	0.59290 (15)	0.50826 (15)	0.16851 (13)	0.0408 (5)	
O3	0.22138 (17)	0.63806 (19)	−0.04018 (17)	0.0591 (6)	
O4	0.70129 (17)	0.70006 (18)	0.19471 (16)	0.0554 (6)	
O5	0.52911 (16)	0.34179 (17)	−0.01527 (15)	0.0418 (5)	
C1	0.2173 (3)	0.3281 (3)	0.0021 (2)	0.0491 (8)	
C2	0.2562 (2)	0.4425 (3)	0.0042 (2)	0.0401 (7)	
C3	0.1772 (3)	0.5299 (3)	−0.0450 (2)	0.0507 (8)	
C4	0.0662 (3)	0.5050 (4)	−0.0929 (3)	0.0706 (11)	
H4	0.0157	0.5640	−0.1236	0.085*	
C5	0.0303 (3)	0.3914 (4)	−0.0949 (3)	0.0858 (14)	
H5	−0.0440	0.3742	−0.1287	0.103*	
C6	0.1032 (3)	0.3068 (4)	−0.0480 (3)	0.0694 (11)	
H6	0.0778	0.2319	−0.0485	0.083*	
C7	0.2873 (3)	0.2305 (3)	0.0488 (2)	0.0492 (8)	
H7	0.2537	0.1606	0.0496	0.059*	
C8	0.4581 (3)	0.1297 (3)	0.1400 (2)	0.0559 (9)	
H8A	0.4930	0.0987	0.0988	0.067*	
H8B	0.4121	0.0699	0.1481	0.067*	
C9	0.5436 (3)	0.1685 (3)	0.2447 (2)	0.0560 (9)	
H9A	0.5097	0.1823	0.2911	0.067*	
H9B	0.5989	0.1089	0.2748	0.067*	
C10	0.6950 (3)	0.2982 (3)	0.2888 (2)	0.0508 (9)	
H10	0.7354	0.2413	0.3357	0.061*	
C11	0.7511 (2)	0.4034 (3)	0.2882 (2)	0.0463 (8)	
C12	0.6982 (2)	0.5033 (3)	0.2313 (2)	0.0390 (7)	
C13	0.7612 (2)	0.6054 (3)	0.2452 (2)	0.0477 (8)	
C14	0.8727 (3)	0.6055 (3)	0.3066 (3)	0.0651 (10)	
H14	0.9134	0.6722	0.3122	0.078*	
C15	0.9243 (3)	0.5060 (4)	0.3601 (3)	0.0777 (12)	
H15	0.9995	0.5065	0.4012	0.093*	
C16	0.8658 (3)	0.4077 (4)	0.3528 (3)	0.0674 (11)	
H16	0.9012	0.3425	0.3906	0.081*	
C17	0.1503 (3)	0.7298 (3)	−0.0960 (3)	0.0694 (11)	
H17A	0.0975	0.7450	−0.0685	0.083*	
H17B	0.1107	0.7091	−0.1685	0.083*	
C18	0.2193 (4)	0.8354 (3)	−0.0848 (3)	0.0903 (14)	
H18A	0.2598	0.8538	−0.0128	0.135*	
H18B	0.1728	0.8995	−0.1199	0.135*	
H18C	0.2693	0.8205	−0.1147	0.135*	
C19	0.7526 (3)	0.8113 (3)	0.2181 (3)	0.0670 (11)	
H19A	0.8031	0.8193	0.1869	0.080*	
H19B	0.7931	0.8209	0.2924	0.080*	
C20	0.6637 (4)	0.8998 (3)	0.1749 (3)	0.0911 (14)	
H20A	0.6247	0.8898	0.1013	0.137*	
H20B	0.6952	0.9759	0.1897	0.137*	
H20C	0.6140	0.8906	0.2060	0.137*	
H5B	0.4751 (16)	0.367 (3)	−0.0680 (17)	0.080*	
H5A	0.5844 (15)	0.379 (3)	−0.013 (2)	0.080*	

### Atomic displacement parameters (Å^2^)

**Table:**

	*U*^11^	*U*^22^	*U*^33^	*U*^12^	*U*^13^	*U*^23^
Zn1	0.0370 (2)	0.0343 (2)	0.0395 (2)	0.00106 (17)	0.01491 (15)	0.00468 (17)
N1	0.0494 (17)	0.0389 (15)	0.0484 (15)	0.0003 (13)	0.0222 (13)	0.0062 (13)
N2	0.0522 (17)	0.0392 (15)	0.0417 (14)	0.0046 (13)	0.0205 (13)	0.0086 (12)
O1	0.0320 (12)	0.0407 (12)	0.0528 (12)	−0.0006 (10)	0.0130 (10)	−0.0009 (10)
O2	0.0332 (11)	0.0366 (11)	0.0417 (11)	0.0014 (9)	0.0061 (9)	0.0019 (10)
O3	0.0442 (13)	0.0526 (15)	0.0689 (15)	0.0142 (12)	0.0137 (11)	0.0076 (13)
O4	0.0525 (14)	0.0398 (13)	0.0657 (14)	−0.0078 (12)	0.0180 (12)	−0.0060 (12)
O5	0.0433 (12)	0.0382 (12)	0.0430 (12)	0.0033 (10)	0.0177 (10)	0.0028 (10)
C1	0.043 (2)	0.057 (2)	0.0493 (19)	−0.0085 (18)	0.0224 (16)	−0.0040 (17)
C2	0.0330 (17)	0.052 (2)	0.0360 (16)	−0.0010 (16)	0.0156 (14)	−0.0049 (15)
C3	0.0376 (19)	0.061 (2)	0.0492 (18)	0.0028 (18)	0.0148 (15)	−0.0022 (18)
C4	0.037 (2)	0.087 (3)	0.076 (2)	0.011 (2)	0.0127 (18)	0.003 (2)
C5	0.037 (2)	0.104 (4)	0.099 (3)	−0.014 (3)	0.013 (2)	−0.008 (3)
C6	0.044 (2)	0.080 (3)	0.080 (3)	−0.015 (2)	0.024 (2)	−0.005 (2)
C7	0.054 (2)	0.044 (2)	0.0536 (19)	−0.0126 (18)	0.0268 (17)	−0.0030 (17)
C8	0.072 (2)	0.0368 (19)	0.067 (2)	−0.0026 (19)	0.038 (2)	0.0096 (18)
C9	0.076 (2)	0.0406 (19)	0.052 (2)	0.0062 (19)	0.0281 (19)	0.0165 (17)
C10	0.055 (2)	0.052 (2)	0.0407 (18)	0.0193 (18)	0.0168 (17)	0.0111 (16)
C11	0.0351 (17)	0.058 (2)	0.0403 (17)	0.0097 (16)	0.0109 (14)	0.0052 (16)
C12	0.0360 (18)	0.0469 (19)	0.0335 (15)	0.0027 (15)	0.0144 (14)	−0.0043 (14)
C13	0.0387 (18)	0.058 (2)	0.0406 (17)	−0.0026 (17)	0.0121 (15)	−0.0064 (16)
C14	0.043 (2)	0.085 (3)	0.059 (2)	−0.015 (2)	0.0145 (18)	−0.003 (2)
C15	0.030 (2)	0.115 (4)	0.068 (2)	0.003 (2)	0.0026 (18)	0.013 (3)
C16	0.039 (2)	0.085 (3)	0.064 (2)	0.011 (2)	0.0084 (18)	0.021 (2)
C17	0.067 (2)	0.075 (3)	0.064 (2)	0.033 (2)	0.027 (2)	0.018 (2)
C18	0.102 (3)	0.063 (3)	0.121 (4)	0.026 (3)	0.063 (3)	0.035 (3)
C19	0.082 (3)	0.053 (2)	0.072 (2)	−0.027 (2)	0.039 (2)	−0.014 (2)
C20	0.127 (4)	0.043 (2)	0.118 (3)	−0.006 (3)	0.067 (3)	−0.006 (2)

### Geometric parameters (Å, °)

**Table:**

Zn1—O1	1.9737 (19)	C8—C9	1.513 (4)
Zn1—O2	1.9990 (18)	C8—H8A	0.9700
Zn1—O5	2.040 (2)	C8—H8B	0.9700
Zn1—N2	2.075 (2)	C9—H9A	0.9700
Zn1—N1	2.080 (2)	C9—H9B	0.9700
N1—C7	1.282 (4)	C10—C11	1.435 (4)
N1—C8	1.467 (4)	C10—H10	0.9300
N2—C10	1.279 (4)	C11—C12	1.416 (4)
N2—C9	1.473 (4)	C11—C16	1.424 (4)
O1—C2	1.312 (3)	C12—C13	1.418 (4)
O2—C12	1.317 (3)	C13—C14	1.381 (4)
O3—C3	1.374 (4)	C14—C15	1.391 (5)
O3—C17	1.423 (4)	C14—H14	0.9300
O4—C13	1.367 (3)	C15—C16	1.362 (5)
O4—C19	1.429 (3)	C15—H15	0.9300
O5—H5B	0.843 (10)	C16—H16	0.9300
O5—H5A	0.850 (10)	C17—C18	1.502 (5)
C1—C6	1.417 (4)	C17—H17A	0.9700
C1—C2	1.418 (4)	C17—H17B	0.9700
C1—C7	1.438 (4)	C18—H18A	0.9600
C2—C3	1.417 (4)	C18—H18B	0.9600
C3—C4	1.386 (4)	C18—H18C	0.9600
C4—C5	1.396 (5)	C19—C20	1.494 (5)
C4—H4	0.9300	C19—H19A	0.9700
C5—C6	1.343 (5)	C19—H19B	0.9700
C5—H5	0.9300	C20—H20A	0.9600
C6—H6	0.9300	C20—H20B	0.9600
C7—H7	0.9300	C20—H20C	0.9600
			
O1—Zn1—O2	93.60 (8)	N2—C9—H9A	110.0
O1—Zn1—O5	106.53 (8)	C8—C9—H9A	110.0
O2—Zn1—O5	98.79 (8)	N2—C9—H9B	110.0
O1—Zn1—N2	144.82 (9)	C8—C9—H9B	110.0
O2—Zn1—N2	87.82 (9)	H9A—C9—H9B	108.4
O5—Zn1—N2	107.97 (9)	N2—C10—C11	125.8 (3)
O1—Zn1—N1	89.13 (9)	N2—C10—H10	117.1
O2—Zn1—N1	161.26 (8)	C11—C10—H10	117.1
O5—Zn1—N1	98.18 (9)	C12—C11—C16	118.9 (3)
N2—Zn1—N1	79.43 (10)	C12—C11—C10	123.8 (3)
C7—N1—C8	122.1 (3)	C16—C11—C10	117.2 (3)
C7—N1—Zn1	126.6 (2)	O2—C12—C11	123.9 (3)
C8—N1—Zn1	111.09 (19)	O2—C12—C13	117.9 (3)
C10—N2—C9	120.7 (3)	C11—C12—C13	118.2 (3)
C10—N2—Zn1	125.3 (2)	O4—C13—C14	124.8 (3)
C9—N2—Zn1	113.86 (19)	O4—C13—C12	114.1 (3)
C2—O1—Zn1	128.84 (19)	C14—C13—C12	121.1 (3)
C12—O2—Zn1	127.15 (18)	C13—C14—C15	120.0 (3)
C3—O3—C17	118.4 (3)	C13—C14—H14	120.0
C13—O4—C19	118.5 (2)	C15—C14—H14	120.0
Zn1—O5—H5B	105 (2)	C16—C15—C14	120.7 (3)
Zn1—O5—H5A	114 (2)	C16—C15—H15	119.7
H5B—O5—H5A	106 (2)	C14—C15—H15	119.7
C6—C1—C2	119.1 (3)	C15—C16—C11	120.9 (3)
C6—C1—C7	117.1 (3)	C15—C16—H16	119.5
C2—C1—C7	123.8 (3)	C11—C16—H16	119.5
O1—C2—C3	117.8 (3)	O3—C17—C18	107.8 (3)
O1—C2—C1	124.8 (3)	O3—C17—H17A	110.1
C3—C2—C1	117.4 (3)	C18—C17—H17A	110.1
O3—C3—C4	124.7 (3)	O3—C17—H17B	110.1
O3—C3—C2	113.8 (3)	C18—C17—H17B	110.1
C4—C3—C2	121.5 (3)	H17A—C17—H17B	108.4
C3—C4—C5	120.0 (4)	C17—C18—H18A	109.5
C3—C4—H4	120.0	C17—C18—H18B	109.5
C5—C4—H4	120.0	H18A—C18—H18B	109.5
C6—C5—C4	119.9 (3)	C17—C18—H18C	109.5
C6—C5—H5	120.0	H18A—C18—H18C	109.5
C4—C5—H5	120.0	H18B—C18—H18C	109.5
C5—C6—C1	122.1 (4)	O4—C19—C20	107.1 (3)
C5—C6—H6	118.9	O4—C19—H19A	110.3
C1—C6—H6	118.9	C20—C19—H19A	110.3
N1—C7—C1	125.5 (3)	O4—C19—H19B	110.3
N1—C7—H7	117.3	C20—C19—H19B	110.3
C1—C7—H7	117.3	H19A—C19—H19B	108.5
N1—C8—C9	107.0 (2)	C19—C20—H20A	109.5
N1—C8—H8A	110.3	C19—C20—H20B	109.5
C9—C8—H8A	110.3	H20A—C20—H20B	109.5
N1—C8—H8B	110.3	C19—C20—H20C	109.5
C9—C8—H8B	110.3	H20A—C20—H20C	109.5
H8A—C8—H8B	108.6	H20B—C20—H20C	109.5
N2—C9—C8	108.4 (2)		

### Hydrogen-bond geometry (Å, °)

**Table:**

*D*—H···*A*	*D*—H	H···*A*	*D*···*A*	*D*—H···*A*
O5—H5A···O3^i^	0.85 (1)	2.41 (2)	3.128 (3)	143 (3)
O5—H5A···O1^i^	0.85 (1)	2.03 (2)	2.781 (3)	147 (3)
O5—H5B···O4^i^	0.84 (1)	2.42 (2)	3.104 (3)	139 (3)
O5—H5B···O2^i^	0.84 (1)	1.96 (2)	2.722 (2)	149 (3)

Symmetry codes: (i) −*x*+1, −*y*+1, −*z*.
